# Phenotypic Trait Subdivision Provides New Sight Into the Directional Improvement of *Eucommia ulmoides* Oliver

**DOI:** 10.3389/fpls.2022.832821

**Published:** 2022-04-08

**Authors:** Peng Deng, Yiran Wang, Fengcheng Hu, Hang Yu, Yangling Liang, Haolin Zhang, Ting Wang, Yuhao Zhou, Zhouqi Li

**Affiliations:** ^1^College of Forestry, Northwest A&F University, Yangling, China; ^2^Lveyang County Forest Tree Seedling Workstation, Forestry Bureau of Lveyang County, Lveyang, China; ^3^College of Humanities and Social Development, Northwest A&F University, Yangling, China; ^4^College of Natural Resources and Environment, Northwest A&F University, Yangling, China; ^5^College of Food Science and Engineering, Northwest A&F University, Yangling, China

**Keywords:** *Eucommia ulmoides* Oliver, economic traits, growth traits, genotype × environment (G × E) interaction, traits correlation, environmental factors

## Abstract

*Eucommia ulmoides* Oliver has been used extensively in many fields. To satisfy increasing demand, great efforts must be made to further improve its traits. However, limited information is available on these traits, which is a factor that restricts their improvement. To improve traits directionally, nine clones were assigned to six sites to analyze the effect of different variation sources (the genotype, site, and genotype × environment interaction) on the phenotypic trait. In addition, a mixed linear model was used to assess the contribution of variations. In general, for most traits, the site effect accounted for a larger proportion of the variance, followed by the genotype and genotype × environment interaction effects. All the studied genotypes and sites had a significant effect, indicating that they could be improved by selecting preferable genotypes or cultivation areas, respectively. Interestingly, growth traits or economic traits could be improved simultaneously. Trait performance and stability are necessary when selecting genotypes. Moreover, the discriminating ability of genotypes should be considered in selecting cultivation areas. Annual mean temperature and annual sunshine duration proved to be crucial factors that affected the traits. They were correlated positively with economic traits and leaf yield and correlated negatively with growth traits. These findings contributed to selecting a wider range of cultivation areas. Regarding the genotype × environment interaction effect, there were significant differences only in the gutta-percha content, the total number of leaves, and the chlorogenic acid content. These traits could also be improved by choosing appropriate genotypes for the local environment. The research has provided preliminary data on the main factors that affect the traits of *E. ulmoides* and offered solutions for trait improvement. This information could be a reference for the trait improvement of other plants.

## Introduction

*Eucommia ulmoides* Oliver is an economically important tree native to China (Wuyun et al., 2018). Known for its rubber and medicinal uses, *E*. *ulmoides* has been widely used in many industries (Zhu and Sun, 2018; Wei et al., 2021). It is distributed extensively and grows well where *Hevea brasiliensis* Muell. Arg. cannot be planted (Li et al., 2020). Besides, gutta-percha produced by *E. ulmoides* performs better in corrosion resistance and insulation than rubber produced by *H. brasiliensis* (Chen et al., 2008). These superior properties make *E. ulmoides* a promising alternative or supplemental resource to *H. brasiliensis*, thus mitigating the rubber resource shortage (Wuyun et al., 2018). Therefore, it has been treated as a strategic tree species (Wuyun et al., 2018). *E. ulmoides* has also been used as a traditional Chinese medicine for more than 2,000 years (He et al., 2014). In 2005, its leaves and bark were listed in the “Pharmacopeia of China” (China Pharmacopeia Committee, 2005). In addition, it is used broadly in landscaping and gardens for its stronger adaption and rapid growth rate (Zhang, 1990; Deng et al., 2021). Hence, it is of high economic, ecological, and social value.

The development of *E. ulmoides*-based industries has shown an upward trend. To satisfy their requirements, great improvements must be made in relevant traits. Gutta-percha, chlorogenic acid, and rutin are the main harvest products of *E. ulmoides* (Deng et al., 2021); hence, their contents (economic traits) and leaf yield determine the economic output. Growth traits, such as tree height, diameter at breast height, and ground diameter, can be used to measure the bark yield (Zhang et al., 2002) and growth situation (Li et al., 2014a; Jin et al., 2020). Regarding the improvement of *E. ulmoides*, superior genotypes (Du, 1997; Zhang et al., 2004; Du et al., 2010a,b,c, 2011, 2013a,b, 2014a,b; Du L. et al., 2014) or cultivation areas (Du et al., 2005; Dong et al., 2011; Wu et al., 2019; Wang et al., 2020) have been selected. However, only sectional effects have been studied, an approach that has a limited comprehensive understanding of how traits could be improved. Better improvement effects would require considering these effects simultaneously.

Phenotypic trait variations of plants are affected by the genotype, environment, and genotype × environment (G × E) interaction effects (Leal-Saenz et al., 2020; Jiang et al., 2021; Matsumoto et al., 2021). Superior genotypes could be used to improve traits without increasing the cultivation area, which is encouraging as land is becoming a rare resource (Marcatti et al., 2017). The phenotype is plastic, and there are differences in performance for the same genotype when it is planted in different sites. A great increasement could be obtained in a short time for traits when they are present in favorable sites (Pei et al., 2020). In addition, the relative performance of a genotype still presents differences among sites due to the G × E interaction (Ramburan et al., 2012). Three approaches have been emphasized for trait improvement, including selecting preferable genotypes or cultivation areas (Fabio et al., 2017) or choosing appropriate genotypes for the local environment (Deng et al., 2015). However, it is not known which effect is better for trait improvement. A mixed linear model has often been used to conduct complex genetic analyses (He et al., 2012; Chen et al., 2018). In this study, this approach has been used to subdivide phenotypic trait variations to analyze their principal effects on traits.

In forest tree breeding, multi-trait improvement has been widely used to satisfy diverse production requirements (Carreras et al., 2016). It is important to establish the relationship among the traits of interest to conduct a combined selection among them (Xiao et al., 2021). This approach allows determining whether these traits could be improved simultaneously (Hong et al., 2014). In previous studies, the relationship between growth traits and wood traits has been studied in *Pinus tabuliformis* Carr (Ouyang et al., 2017), *Eucalyptus dunnii* Maiden (Gallo et al., 2018), *Populus ussuriensis* Kom (Jin et al., 2019), *Larix kaempferi* Carriere (Pan et al., 2020), *Casuarina junghuhniana* Miq, and *Casuarina cunninghamiana* Miq (Suraj et al., 2021). This is especially suitable for *E. ulmoides*, as it is a multipurpose tree.

*Eucommia ulmoides* is unique to and widely distributed in China. The natural distribution is mainly concentrated in the area of 25–35°N and 104–119°E. Since its introduction and domestication, it has been cultivated in 27 provinces of China (Wang et al., 2016) with an area of 350,000 hm^2^ (24.5–41.5°N, 76–126°E) (Du, 2014). However, not all distribution areas are optimal for its growth because large differences exist in environmental conditions. In addition, as larger areas are needed, *E. ulmoides* breeding programs are complex and expensive to implement (Souza et al., 2019). Considering that the cultivation area is a comprehensive reflection of various environmental factors (Jiang et al., 2021), it is necessary to analyze the crucial factors that affect the traits. This information would be beneficial in selecting a wider range of cultivation areas. In addition, it lays a theoretical basis for the introduction of *E. ulmoides* to a new region, a factor that would contribute to enlarging the cultivation areas.

Genotype and environment can interact mutually, which shows the adaptation of a genotype to the environment. The G × E interaction effect leads to differences in adaption. For example, genotypes that perform better in one environment may perform inferiorly in other environments (Li et al., 2015). Therefore, a clear understanding of the G × E interaction effect is crucial for *E. ulmoides* improvement (Jiang et al., 2021). Researchers have used the genotype + genotype × environment (GGE) interaction biplot (Yan et al., 2000; Yan, 2010) to analyze the G × E interaction effect in *Populus* (Liu et al., 2020; Jiang et al., 2021). In this approach, groups are divided according to the G × E interaction effect to select appropriate genotypes for the local environment (Frutos et al., 2014).

To better guide *E. ulmoides* directional improvement, nine clones were distributed across six sites to assess the effect and contribution of different variation sources on phenotypic traits.

## Materials and Methods

### Materials and Experimental Design

**Figure 1:**
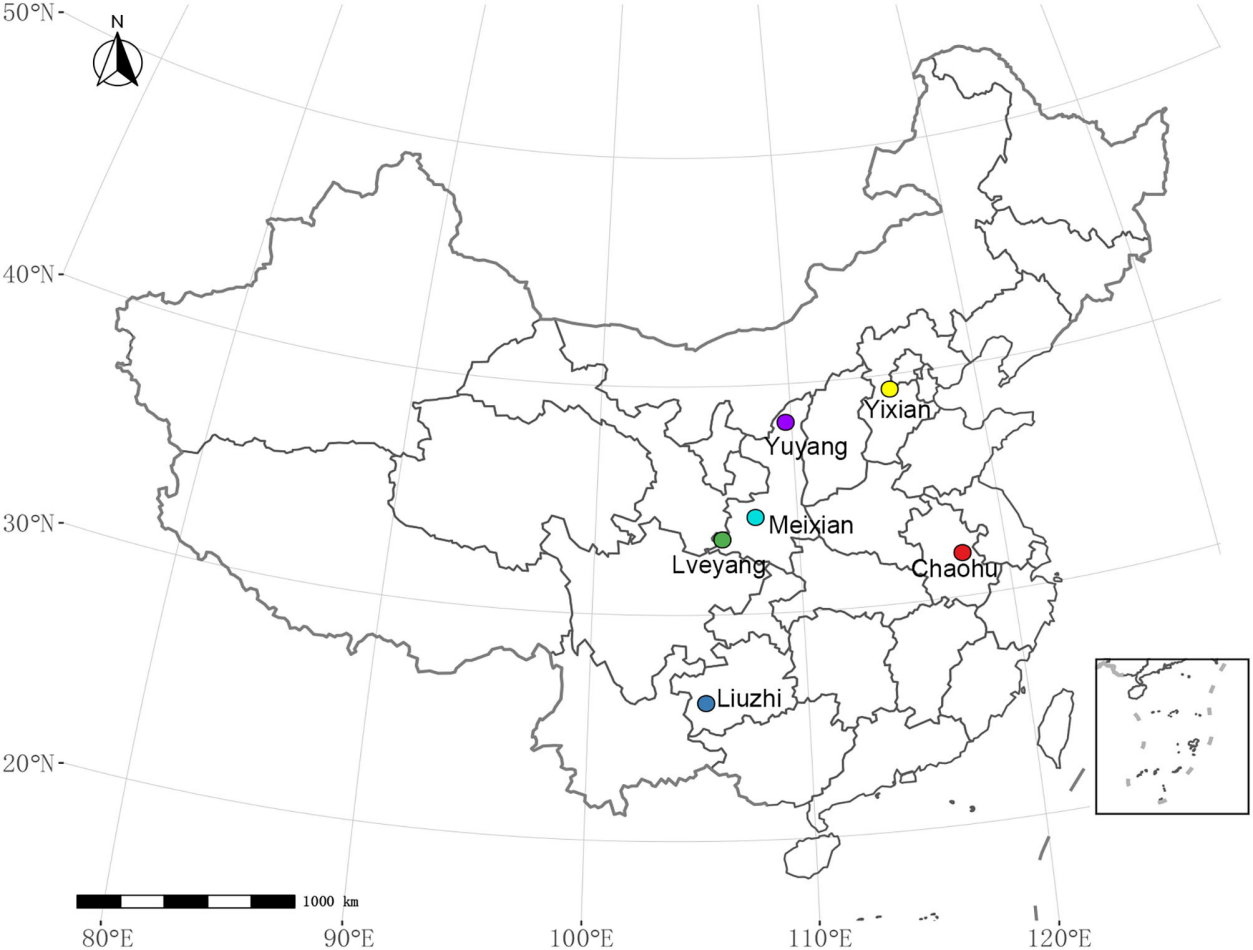
Distribution of the experimental sites.

A total of 9 *E. ulmoides* clones were employed in this study, which included two selected clones and seven hybrid clones (Table 1). The two selected clones were authorized cultivars; they were selected from 40 clones from all over China (Zhang et al., 2004). The seven hybrid clones were selected from the hybrid offspring of superior varieties (Li et al., 2014b) in China according to secondary metabolite content and growth traits. The nine clones were grafted with the same 1-year-old *E. ulmoides* clones on the trunk near the root. The purpose of grafting is to eliminate the differences among individuals. They were assigned to six sites (Figure 1, Supplementary Figure 1) throughout China in November 2019. The main environmental factors of experimental sites are listed in Table 2. A total of 9 clones were laid out in a completely randomized block design, including three blocks with six individuals per block at a spacing of 2.0 × 3.0 m. To maintain the relative consistency of the growing environment, protective row seedlings at similar heights were arranged around the tested seedlings with equal spacing. In addition, weeding was conducted once in the summer and once in the autumn, and other measures, such as irrigation and fertilization, were not applied.

**Table 1:** The sources of the clones.

**Clone**	**Abbr**.	**Origin of clone**	**Parental combination**	**Sex**
Clone 2	2	Hybrid	“Xiaoye” × “Qinzhong No. 2”	Male
Clone 3	3	Hybrid	“Xiaoye” × “Luochao No. 3”	Female
Clone 4	4	Hybrid	“Xiaoye” × “Ziye”	Male
Clone 5	5	Hybrid	“Xiaoye” × “Longguai”	Male
Clone 16	16	Hybrid	“Yanci” × “Qinzhong No. 1”	Female
Clone 17	17	Hybrid	“Yanci” × “Qinzhong No. 2”	Female
Clone 22	22	Hybrid	“Huazhong No. 2” × “Qinzhong No. 2”	Male
Qinzhong No. 3	Q3	Excellent natural individual	\	Female
Qinzhong No. 4	Q4	Excellent natural individual	\	Female

*Abbr., Abbreviation of the clones*.

**Table 2:** The main environmental factors and related data of six experimental sites throughout China.

**Experimental site**	**Longitude and Latitude**	**Soil type**	**Altitude (m)**	**AMT (**°**C)**	**AAP (mm)**	**ASD (h)**	**RH (%)**
Chaohu, Anhui	117°50'42.4”E 31°52'54.5”N	Yellow brown soil	64	16.4	1124.5	1893.3	75
Liuzhi, Guizhou	105°20'22.4”E 26°6'18.0”N	Yellow soil	1,437	14.8	1,480	1623.6	80
Yixian, Hebei	115°20'35”E 39°21'28”N	Brown soil	92	12.5	538.7	2358	62
Yuyang, Shaanxi	109°41'59.4”E 38°19'19.9”N	Moisture soil	1,068	8.8	383.6	2,684	54
Meixian, Shaanxi	107°54'21.2”E 34°13'51.7”N	Cinnamon soil	423	12.8	581.6	1739.6	74
Lveyang, Shaanxi	106°14'29.8”E 33°17'39.0”N	Brown soil	1,196	13.6	777.6	1,493	72

*AMT, annual mean temperature; AAP, annual average precipitation; ASD, annual sunshine duration; RH, relative humidity*.

### Sample Processing and Growth Traits Measurement

For each clone, five pieces of mature fresh leaves (the 8th to 12th leaves from the top) were collected from all the seedlings in each block in September 2020. The samples were mixed fully and placed in a drying oven at 60°C. Then, they were stored hermetically at room temperature for later use. Growth traits were measured after the leaves fell in 2020. Tree height was measured by using a box staff. A Vernier caliper (111N-101-10G, Guilin Guanglu Measuring Instrument Co., Ltd.) was used to measure ground diameter and diameter at breast height. The presented phenotypic trait values are the averages of the three blocks.

### Economic Trait Measurement

The samples were ground into powder by using a tissue grinder apparatus (DHS TL2020, DHS Life Science & Technology Co., Ltd.) before measuring economic traits.

The extraction and determination of gutta-percha were performed according to the description of Ma et al. (1994). In brief, 100 ml of NaOH (Guangdong Guanghua Sci-Tech Co., Ltd.) solution (10%, m/m) was added to about 5 g of powder selected randomly from each block and heated in a water bath at 90°C for 3 h (two times total). After filtration, 60 ml of HCl (Xilong Scientific Co., Ltd.) was added to the residue in the water bath at 40°C for 2 h. Then, 60 ml of ethanol (Guangdong Guanghua Sci-Tech Co., Ltd.) (60%, v/v) was poured into the residue after filtration, holding for 1 h, and processed in an ultrasonic cleaner (KQ-600DV, Kunshan Ultrasonic Equipment Company) (40 kHz, 40°C) for 0.5 h. After filtration and drying at room temperature, gutta-percha was obtained. The gutta-percha content is presented as the average content of three blocks.

Extraction of effective medicinal components (chlorogenic acid and rutin) was conducted according to the study by Dong et al. (2011). The components were separated as described by Ye et al. (2019), and determination was performed *via* a high-performance liquid chromatography (HPLC) system (1260 Infinity II, Agilent Technologies, Inc.). Chlorogenic acid and rutin were detected at 320 and 360 nm, respectively. The 1260 DAD-chemstation (off-line) software was used for data analysis. Chlorogenic acid and rutin concentrations were calculated based on external calibration by peak areas. They are presented as the average of the contents in three blocks. Chlorogenic acid (HPLC ≥ 98%) and rutin (HPLC ≥ 98%) standards were purchased from Shanghai Yuanye Bio-Technology Co., Ltd.

### Data Sources and Analysis

#### Acquiring the Environmental Data Related to the Experimental Sites

The longitude, latitude, and altitude were obtained using a handheld GPS locator (Caitu N130, BHC Navigation Co., Ltd.). The environmental factor data (Table 2) were obtained from the stations closest to the experimental sites in the China Meteorological Data Service Center (http://data.cma.cn/en).

#### Chlorogenic Acid and Rutin Content Determination

The chlorogenic acid and rutin content were determined from the following standard curves:


(1) $$\begin{array}{r} \text{Chlorogenic acid content: }y\;=\;2\times 10^{-5}x+0.0041\\ \left(R^{2}\;=\;0.9992\right) \end{array}$$

(2) $$\begin{array}{l} \text{Rutin content: }y\;=\;4\times 10^{-5}x-0.0026\;\left(R^{2}\;=\;0.9991\right) \end{array}$$

In Equations (1) and (2), *x* is the peak area and *y* is the content.

#### The Mixed Linear Model for Traits Analysis

The universal mixed linear model was as follows:


(3) $$\begin{array}{l} y\;=\mu +S_{i}+\;G_{j}+{SG}_{ij}\;+\;e_{ijk} \end{array}$$

In Equation (3), μ is the overall average, *S*_*i*_ is the site effect, *G*_*j*_ is the genotype effect, *SG*_*ij*_ is the interaction effect between the site and genotype, and e_ijk_ is the error effect. All the effects were random.

#### Normal Distribution Test, Variance Analyses, and Multiple Comparisons

The Shapiro–Wilk test was used to check the normality of the data. The *F*-test of variance analysis in R was used to test for the significance of the effects. Multiple comparisons were conducted using Duncan's test. The ASReml-R V4.0 statistical analysis package (Gilmour et al., 2009) was used to estimate the variance component of traits.

#### Trait Correlation Analysis

The genetic (phenotypic) correlation among traits was obtained by using the ASReml-R version 4.0 statistical analysis package (Gilmour et al., 2009) by using the following expression:

(4) $$\begin{array}{l} r_{ij}=\frac{V_{ij}}{sqrt\left(V_{i}*V_{j}\right)} \end{array}$$

In Equation (4), *r*_*ij*_ is the genetic (phenotypic) correlation coefficient between trait *i* and *j, V*_*ij*_ is the covariance between trait *i* and *j*, and *V*_*i*_ and *V*_*j*_ are the genetic (phenotypic) variance estimated for trait *i* and *j*, respectively.

#### Gray Correlation Analysis

In Microsoft Office Excel 2016, homogenization was used to process dimensionless data in gray correlation analyses among traits and environmental factors.

#### Path Analysis

Path coefficient analyses among traits and environmental factors were conducted by using linear regression in IBM SPSS Statistics 22 (Du and Chen, 2010).

#### Data Visualization

R software was used to create all the figures. The “ggplot2” package was used to create the map of the experimental sites, the variance component of the traits, correlations among traits, and boxplots. The “pheatmap” package was used to create the heat map. The “GGEBiplotGUI” package was used to create the biplot.

## Results

### Subdivision Based on Phenotypic Traits

It was obvious that all the studied traits were affected significantly by the site and genotype effects (Table 3). In other words, the improvement of the studied traits could depend on selecting cultivation areas or genotypes. In addition, there were extremely significant differences for the G × E interaction effect only in the gutta-percha content, the chlorogenic acid content, and the total number of leaves. These traits could also be improved by selecting appropriate genotypes in the local environment.

**Table 3:** Variance analysis of traits.

**Trait**	***P*-value**	**Site effect**				**Genotype effect**				**G × E interaction effect**				**Error effect**	
		**Df**	**MS**	***F***	***P***	**Df**	**MS**	***F***	***P***	**Df**	**MS**	***F***	***P***	**Df**	**MS**
Gutta-percha content	0.072	5	0.9415	21.296	0.000	8	0.7151	16.173	0.000	40	0.0969	2.191	0.001	108	0.0442
Chlorogenic acid content	0.212	5	6.979	34.871	0.000	8	1.663	8.310	0.000	40	0.307	1.536	0.042	108	0.200
Rutin content	0.619	5	0.2022	32.15	0.000	8	0.1084	17.24	0.000	40	0.0081	1.29	0.152	108	0.0063
Total number of leaves	0.368	5	5.040	41.043	0.000	8	4.060	33.061	0.000	40	0.411	3.344	0.000	824	0.123
Tree height	0.086	5	38,828	50.87	0.000	8	32,559	42.66	0.000	40	626	0.82	0.78	874	763
Diameter at breast height	0.329	5	367.5	53.554	0.000	8	41.3	6.021	0.000	40	7.3	1.065	0.364	864	6.9
Ground diameter	0.071	5	606.2	82.741	0.000	8	41.8	5.705	0.000	40	5.9	0.799	0.809	874	7.3

*p-value, p-value of the Shapiro–Wilk normality test; Df, degrees of freedom; MS, mean square; F, F value; P, P-value of analysis of variance*.

**Figure 2:**
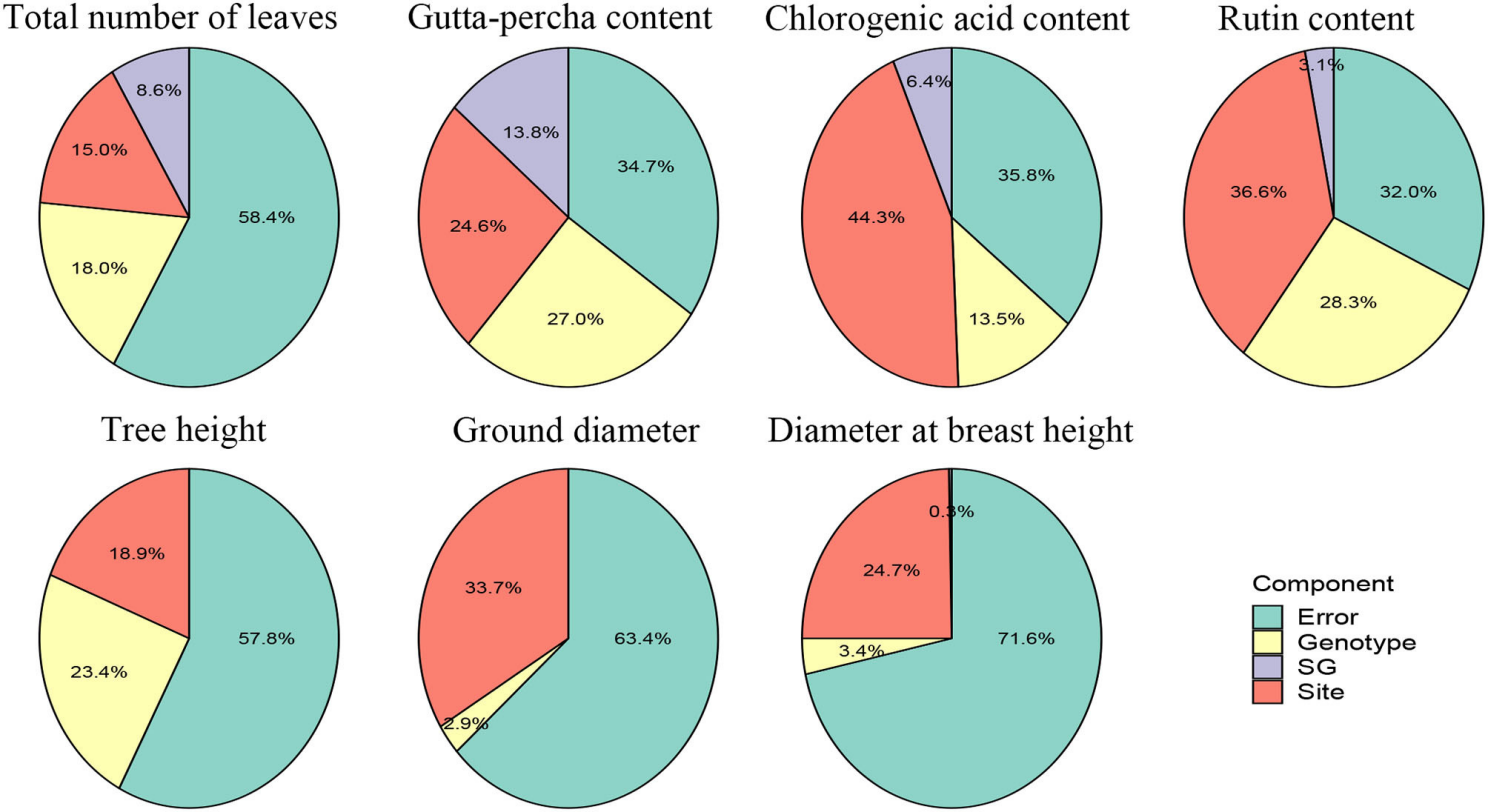
Variance component proportions of traits.

To illustrate the relative importance of the effects, the proportions of the variance components for the traits are presented in Figure 2. Although there were big differences among the traits for the proportion of each effect, the relative importance of the effects was similar. In general, the site effect accounted for a larger proportion of the variance, followed by the genotype and G × E interaction effects. For the total number of leaves, the gutta-percha content, and tree height, the genotype effect took a relatively major proportion compared with the site effect. Furthermore, for growth traits, except for tree height, the genotype and G × E interaction effects contributed relatively less to the variances, indicating they could be improved mainly depending on selecting cultivation areas. Regarding economic traits, the error effect contributed less to the variance compared with growth traits, which showed their greater stability.

### Effect of Genotypes on Traits

#### Multiple Comparisons of the Traits Among Genotypes

**Figure 3:**
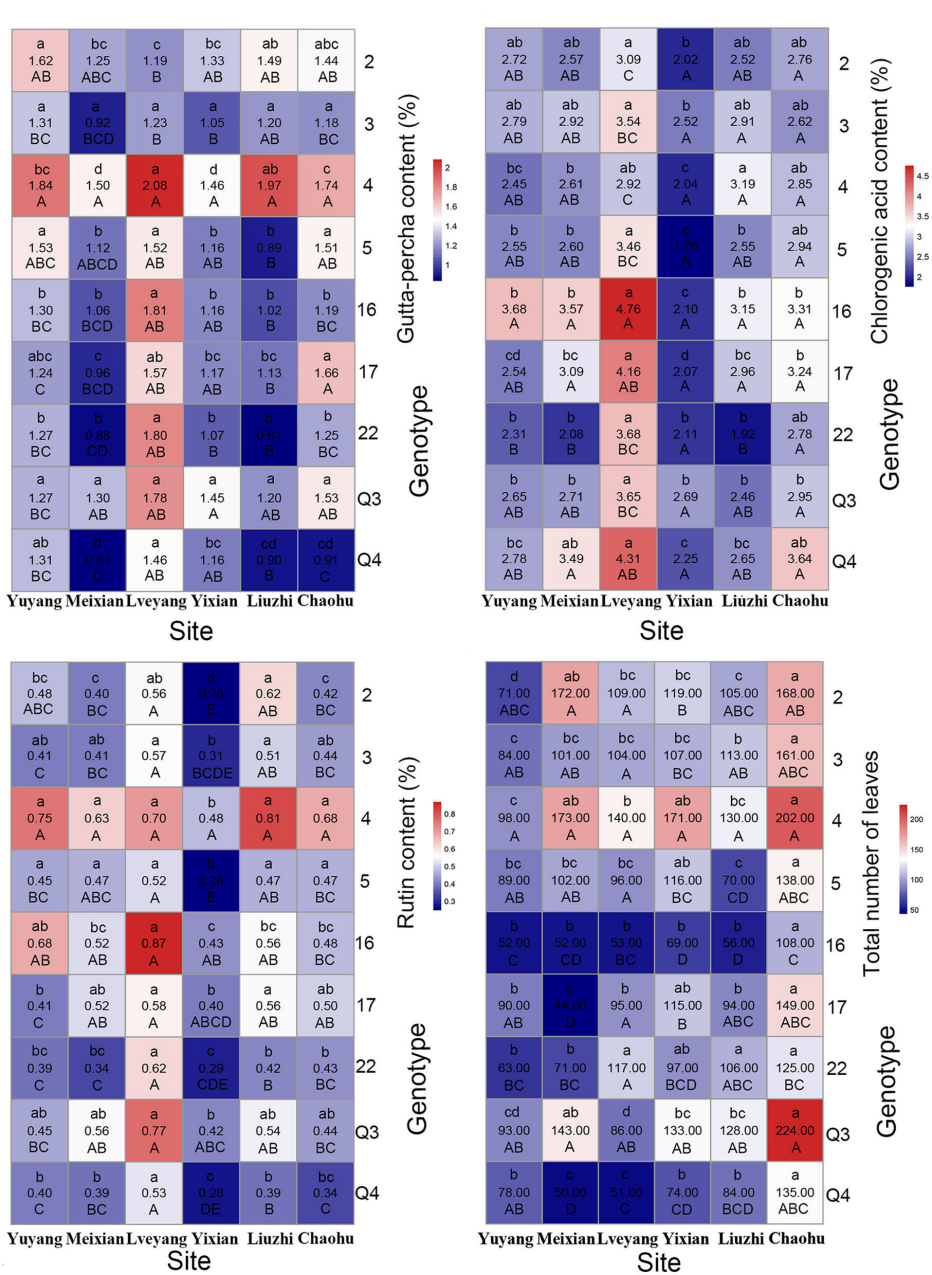
Mean heat map of economic traits and leaf yield. For each grid, the number in the middle indicates the trait mean of a clone at a site. The capital letter below the number indicates the multiple comparison results among clones for a certain site. The lowercase letter above the number indicates the multiple comparison results among the sites for a certain clone.

**Figure 4:**
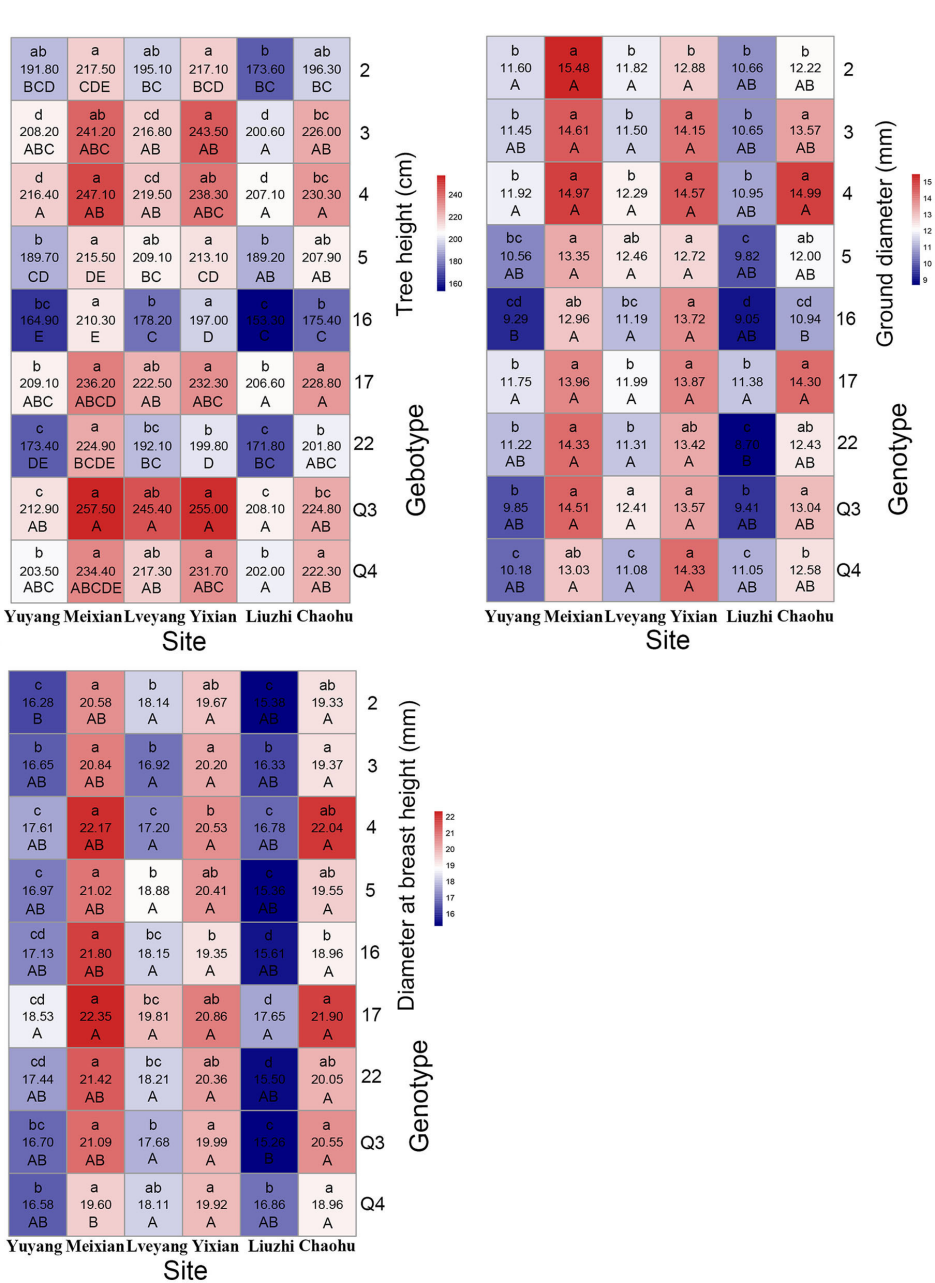
Mean heat map of growth traits (refer to the details in the Figure 3 legend).

Genotypes had a significant effect on the studied traits. Multiple comparison results revealed distinct features for different traits (Table 4). Clone 4 performed best in the gutta-percha content, rutin content, tree height, diameter at breast height, ground diameter, and the total number of leaves among (Table 4) or within (Figures 3, 4) the experimental sites. The highest effective medicinal component contents were found in clone 16 among (Table 4) or within (Figure 3) the experimental sites, except for the rutin content in Chaohu. Clone 17 had the best growth traits among (Table 4) or within (Figure 4) the experimental sites. All three clones were significantly different from other genotypes for the corresponding traits, which showed their better performance. Considering that a preferable clone must be abundant in yield and excellent in stability, differences among genotypes were studied for the variation range. The variation range of clones 4 and 17 was obviously smaller among cultivation areas for corresponding traits, while that of clone 16 was larger, indicating that clones 4 and 17 were more stable across sites (Table 4). In conclusion, for traits that are mainly improved by selecting genotypes, trait performance and stability should be considered.

**Table 4:** Multiple comparisons for traits among genotypes.

**Genotype**	**Gutta-percha content (%)**		**Chlorogenic acid content (%)**		**Rutin content (%)**		**Total number of leaves**	
	***X* ± *S^a^***	**CV (%)**	***X* ± *S^a^***	**CV (%)**	***X* ± *S^a^***	**CV (%)**	***X* ± *S^a^***	**CV (%)**
2	1.39 ± 0.19 B	13.67	2.61 ± 0.50 BC	19.16	0.46 ± 0.13 CD	28.26	118.64 ± 54.13 BC	45.63
3	1.15 ± 0.22 BC	19.13	2.89 ± 0.55 ABC	19.03	0.44 ± 0.12 CD	27.27	111.32 ± 42.15 BCD	37.86
4	1.77 ± 0.25 A	14.12	2.68 ± 0.44 BC	16.42	0.67 ± 0.14 A	20.90	149.27 ± 67.28 A	45.07
5	1.29 ± 0.28 BC	21.71	2.64 ± 0.61 BC	23.11	0.44 ± 0.11 CD	25.00	100.60 ± 43.76 CD	43.50
16	1.26 ± 0.35 BC	27.78	3.43 ± 0.92 A	26.82	0.59 ± 0.18 AB	30.51	65.81 ± 36.71 E	55.78
17	1.29 ± 0.32 BC	24.81	3.01 ± 0.72 ABC	23.92	0.50 ± 0.09 BCD	18.00	99.46 ± 54.67 D	54.97
22	1.18 ± 0.39 BC	33.05	2.48 ± 0.79 C	31.85	0.42 ± 0.13 CD	30.95	97.86 ± 55.51 D	56.72
Q3	1.42 ± 0.33 B	23.24	2.85 ± 0.48 ABC	16.84	0.53 ± 0.18 BC	33.96	129.70 ± 70.46 AB	54.33
Q4	1.10 ± 0.27 C	24.55	3.19 ± 0.85 AB	26.65	0.39 ± 0.09 D	23.08	78.55 ± 47.20 E	60.09
Average		22.45		22.64		26.44		50.44
**Genotype**	**Tree height (cm)**		**Diameter at breast height (mm)**		**Ground diameter (mm)**			
	***X*** ± ***S**^a^*	**CV (%)**	***X*** ± ***S**^a^*	**CV (%)**	***X*** ± ***S**^a^*	**CV (%)**		
2	198.84 ± 36.73 C	18.47	12.49 ± 3.42 AB	27.38	18.24 ± 3.36 B	18.42		
3	222.60 ± 28.66 AB	12.88	12.67 ± 2.44 AB	19.26	18.41 ± 3.17 B	17.22		
4	226.50 ± 22.55 AB	9.96	13.31 ± 2.55 A	19.16	19.51 ± 3.03 AB	15.53		
5	204.46 ± 32.44 C	15.87	11.84 ± 3.06 BC	25.84	18.77 ± 3.32 B	17.69		
16	180.18 ± 28.80 D	15.98	11.22 ± 3.21 C	28.61	18.54 ± 3.13 B	16.88		
17	222.48 ± 24.48 AB	11.00	12.88 ± 2.46 AB	19.10	20.17 ± 2.86 A	14.18		
22	195.14 ± 33.69 C	17.26	12.10 ± 3.56 BC	29.42	19.04 ± 3.76 AB	19.75		
Q3	233.98 ± 41.42 A	17.70	12.16 ± 3.38 ABC	27.80	18.57 ± 3.68 B	19.82		
Q4	219.05 ± 27.13 B	12.39	12.07 ± 2.55 BC	21.13	18.41 ± 2.79 B	15.15		
Average		14.61		24.19		17.18		

a *The same letter for the same trait indicates an insignificant difference, while a different letter indicates a very significant difference (P < 0.01). “X,” “S,” and “CV” indicate trait average, standard, and coefficient of variation, respectively, of a clone at six sites; “Average,” the average CV of all the clones*.

#### Correlations Among Traits

**Figure 5:**
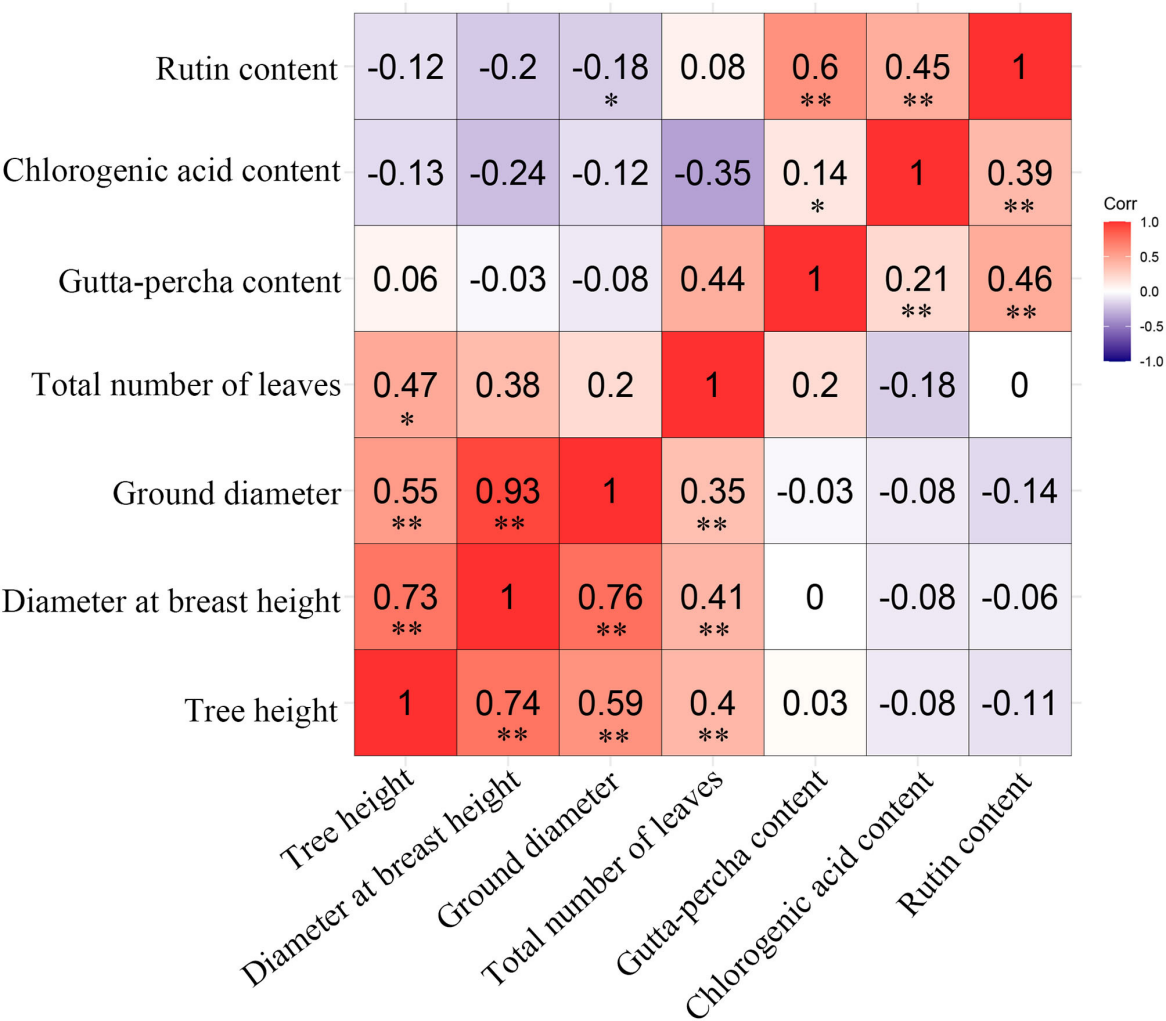
Genetic (above diagonal) and phenotypic (below diagonal) correlations among traits. “**” and “*” represent a very significant difference (*P* < 0.01) and a significant difference (*P* < 0.05), respectively.

Because different traits showed their best performance in different genotypes, the preferable genotypes for specific traits were selected. However, the selected clones performed better only in certain aspects, which is a phenomenon that would not meet the requirements of comprehensive utilization. Therefore, simultaneous multi-trait improvement is necessary, and this approach needs to be guided by the correlations among traits. There were similar results in genetic and phenotypic correlation among the studied traits (Figure 5). There were significant positive correlations for traits within growth or economic traits. However, there was a weak negative correlation for traits among growth and economic traits. Fortunately, the gutta-percha content and tree height were correlated positively. It could be concluded that the traits within growth or economic traits could be improved simultaneously, while the traits among growth and economic traits could not be improved simultaneously. The total number of leaves was correlated positively with growth and economic traits except for the chlorogenic acid content, indicating leaf yield could be improved simultaneously with nearly all the studied traits.

#### Effect of Sex on Traits

**Figure 6:**
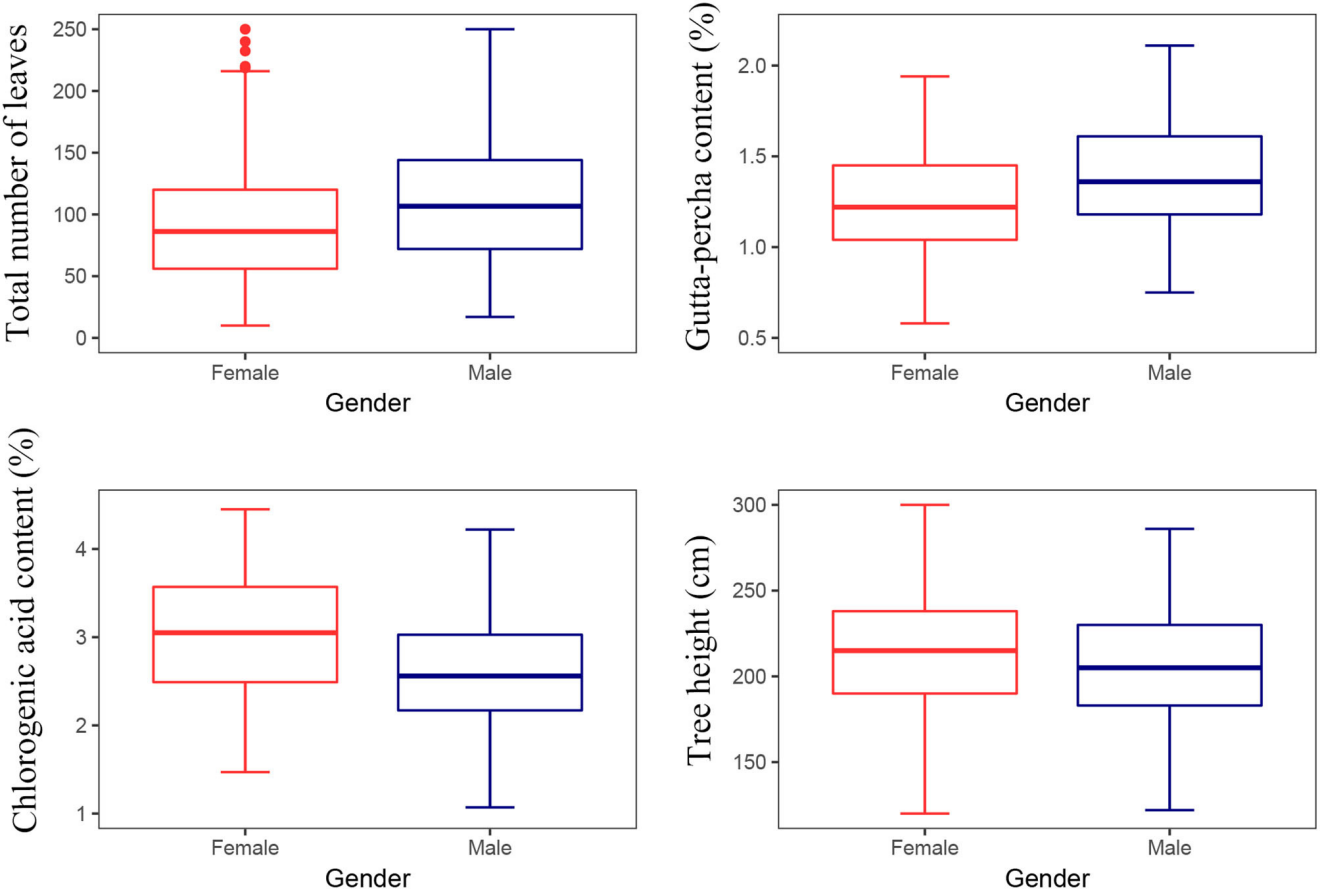
Multiple comparisons for traits between the sexes.

As a dioecious plant, the sex difference is an important component of the genotype difference for *E. ulmoides*. There were significant differences between the sexes for the gutta-percha content, the chlorogenic acid content, the total number of leaves, and tree height (Table 5). In particular, the chlorogenic acid content and tree height of female trees were greater than those of male trees, while the gutta-percha content and the total number of leaves were greater for male trees (Figure 6).

**Table 5:** The significant test of the sex effect.

**Trait**	**Sex**				**Error**	
	**Df**	**MS**	***F*-value**	***P*-value**	**Df**	**MS**
Gutta-percha content	1	1.0835	9.634	0.002	160	0.1125
Chlorogenic acid content	1	8.808	19.23	0.000	160	0.458
Rutin content	1	0.000105	0.006	0.939	160	0.018011
Total number of leaves	1	6.394	33.16	0.000	876	0.193
Tree height	1	18,847	15.47	0.000	926	1,218
Diameter at breast height	1	12.652	1.383	0.240	916	9.145
Ground diameter	1	0.919	0.085	0.771	926	10.801

*Df, degrees of freedom; MS, mean square*.

### Effect of Cultivation Areas on Traits

The site had a significant effect on traits, and it accounted for a larger proportion of variation, which could be treated as an alternative to trait improvement. To better guide trait improvement, the differences in traits among cultivation areas were studied.

#### Multiple Comparisons of the Traits Among Cultivation Areas

As shown in Table 6, multiple comparison results among sites varied from trait to trait. Considering all the clones, the highest economic component contents occurred in Lveyang and were significantly higher than the other sites. When considering each clone as a research unit (Figure 3), the highest economic component contents also occurred in Lveyang, except for clone 2 regarding the gutta-percha content. The results demonstrated that Lveyang was more suitable for the accumulation of the economic component contents and could be a candidate area to improve economic traits. Multiple comparison analyses of the chlorogenic acid and rutin contents among sites were consistent, and sites could be classified into three grades. Lveyang was in the first grade (a), with the highest content. Yixian was in the third grade (c), with the lowest content. Other sites were in the second grade (b), with an intermediate content. It seems that the chlorogenic acid and rutin contents could be improved concurrently by selecting cultivation areas. Although there were differences in the multiple comparison results among growth traits, the relative performance of cultivation areas was consistent. Meixian performed the best, followed by Yixian, Chaohu, Lveyang, Yuyang, and Liuzhi. For the total number of leaves, Chaohu and Yixian performed the best, followed by Liuzhi, Meixian, Lveyang, and Yuyang.

**Table 6:** Multiple comparisons for traits among the experimental sites.

**Site**	**Gutta-percha content (%)**		**Chlorogenic acid content (%)**		**Rutin content (%)**		**Total number of leaves**	
	***X* ± *S^a^***	**CV (%)**	***X* ± *S^a^***	**CV (%)**	***X* ± *S^a^***	**CV (%)**	***X* ± *S^a^***	**CV (%)**
Yuyang	1.41 ± 0.23 b	16.31	2.72 ± 0.56 b	20.59	0.49 ± 0.14 b	28.57	80.02 ± 36.09 d	45.10
Meixian	1.09 ± 0.25 d	22.94	2.85 ± 0.56 b	19.65	0.47 ± 0.11 b	23.40	97.00 ± 60.24 cd	62.10
Lveyang	1.61 ± 0.36 a	22.36	3.73 ± 0.63 a	16.89	0.64 ± 0.18 a	28.13	94.43 ± 54.40 cd	57.61
Yixian	1.22 ± 0.18 cd	14.75	2.17 ± 0.58 c	26.73	0.35 ± 0.09 c	25.71	110.31 ± 51.56 b	46.74
Liuzhi	1.18 ± 0.44 d	37.29	2.70 ± 0.49 b	18.15	0.54 ± 0.15 b	27.78	99.55 ± 43.07 bc	43.26
Chaohu	1.38 ± 0.29 bc	21.01	3.01 ± 0.47 b	15.61	0.47 ± 0.11 b	23.40	153.32 ± 69.38 a	45.25
Average		22.44		19.60		26.17		50.01
**Site**	**Tree height (cm)**		**Diameter at breast height (mm)**		**Ground diameter (mm)**			
	***X*** ± ***S**^a^*	**CV (%)**	***X*** ± ***S**^a^*	**CV (%)**	***X*** ± ***S**^a^*	**CV (%)**		
Yuyang	196.47 ± 28.03 c	14.27	10.87 ± 2.39 d	21.99	17.10 ± 2.17 d	12.69		
Meixian	231.42 ± 29.64 a	12.81	14.10 ± 2.77 a	19.65	21.20 ± 2.76 a	13.02		
Lveyang	210.46 ± 36.63 b	17.40	11.79 ± 2.67 c	22.65	18.28 ± 3.03 c	16.58		
Yixian	225.06 ± 35.08 a	15.59	13.68 ± 2.57 a	18.79	20.13 ± 2.74 b	13.61		
Liuzhi	190.58 ± 28.11 c	14.75	10.27 ± 2.40 d	23.37	16.12 ± 2.39 e	14.83		
Chaohu	212.35 ± 34.48 b	16.24	12.90 ± 3.18 b	24.65	20.09 ± 3.28 b	16.33		
Average		15.18		21.85		14.51		

a *The same letter for the same trait indicates an insignificant difference, while a different letter indicates a significant difference (P < 0.05). “X,” “S,” and “CV” indicate trait average, standard, and coefficient of variation, respectively, of all clones at an experimental site; “Average,” the average CV in all experimental sites*.

In sum, the studied traits could be improved by selecting cultivation areas. Although different traits had their best performance in different cultivation areas, approximately the same results were obtained for economic or growth traits.

#### Discriminating Ability of Cultivation Areas on Genotypes

The effect of cultivation areas on traits could also discriminate among genotypes, which is a consideration for genotype selection. For each trait, the coefficient of variation of each site stands for the ability to discriminate among genotypes. In general, there were big differences among cultivation areas in the discriminating ability of the studied traits (Table 6). Liuzhi and Meixian performed better in distinguishing genotypes for the gutta-percha content; Yuyang performed better in distinguishing genotypes for the chlorogenic acid and rutin contents; Lveyang and Chaohu performed better in distinguishing genotypes for growth traits; and Meixian and Lveyang performed better in distinguishing genotypes for the total number of leaves. These findings could be a reference for genotype selection, which benefits trait improvement.

#### The Effect of Environmental Factors on Traits

Although the studied traits could be improved by selecting cultivation areas, limited experimental areas restrict further improvement. Considering that the cultivation area is a comprehensive reflection of various environmental factors, it makes sense to analyze the main environmental factors that affect traits. In this way, a wider range of cultivation areas could be selected according to specific breeding targets, and better improvement could be obtained.

As shown in Table 7, for the gutta-percha content and diameter at breast height, the key environmental factors were annual sunshine duration, followed by annual mean temperature, annual average precipitation, and altitude. For the other traits studied, the major factors were annual mean temperature, followed by annual sunshine duration, annual average precipitation, and altitude. In general, annual mean temperature and annual sunshine duration were the leading environmental factors that affected the traits.

**Table 7:** Gray correlation analysis among traits and environmental factors.

**Trait**	**Gray correlation coefficient**			
	**Altitude**	**AMT**	**AAP**	**ASD**
Gutta-percha content	0.507	0.785	0.634	0.786
Chlorogenic acid content	0.486	0.780	0.630	0.714
Rutin content	0.465	0.756	0.605	0.680
Total number of leaves	0.477	0.868	0.753	0.791
Tree height	0.454	0.831	0.644	0.818
Diameter at breast height	0.428	0.771	0.627	0.794
Ground diameter	0.432	0.790	0.635	0.783

*AMT, annual mean temperature; AAP, annual average precipitation; ASD, annual sunshine duration*.

Although the relative importance of environmental factors to traits was known, the correlation among them was not clear, which could be obtained by path analysis (Table 8). In general, economic traits and leaf yield were correlated negatively with annual average precipitation and correlated positively with other environmental factors. Interestingly, the growth traits were exactly the opposite of the economic traits. Specifically, all the studied environmental factors were correlated very significantly with the gutta-percha content and correlated insignificantly with the total number of leaves. Diameter at breast height and ground diameter were correlated very significantly with altitude and annual sunshine duration, correlated significantly with annual mean temperature, and correlated insignificantly with annual average precipitation.

**Table 8:** Path analysis among traits and environmental factors.

**Trait**	**Altitude**		**AMT**		**AAP**		**ASD**	
	**Path coefficient**	**Sig**.	**Path coefficient**	**Sig**.	**Path coefficient**	**Sig**.	**Path coefficient**	**Sig**.
Gutta-percha content	1.921	0.000	3.262	0.000	−2.337	0.000	1.382	0.000
Chlorogenic acid content	1.502	0.000	2.403	0.001	−1.943	0.000	0.442	0.172
Rutin content	1.095	0.007	1.236	0.086	−0.969	0.055	0.174	0.618
Total number of leaves	0.127	0.747	1.262	0.078	−0.400	0.417	0.594	0.091
Tree height	−0.849	0.033	−0.714	0.310	0.028	0.954	−0.757	0.031
Diameter at breast height	−1.233	0.000	−0.932	0.046	0.169	0.597	−0.881	0.000
Ground diameter	−1.270	0.000	−0.859	0.021	0.143	0.573	−0.877	0.000

*Sig., significance level of the correlation among traits and environmental factors; AMT, annual mean temperature; AAP, annual average precipitation; ASD, annual sunshine duration*.

In summary, statistical analyses indicated that the higher the annual mean temperature and the longer the annual sunshine duration, the higher the economic component contents and leaf yield, but the worse the growth traits. These findings could be regarded as a reference for the selection of a wider range of preferable cultivation areas for specific breeding targets, an approach that could enlarge the cultivation areas and improve traits.

### The Effect of the G × E Interaction on Traits

**Figure 7:**
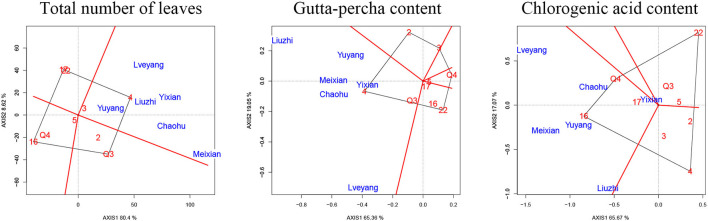
Biplot of the interaction effect. The X-axis and Y-axis are the two principal components. The number or the number and letter combination in red indicates the genotypes of clones. The letter in blue indicates the experimental sites.

There were marked differences in the G × E interaction effect for the studied traits (Figures 3, 4). However, only the gutta-percha content, the total number of leaves, and the chlorogenic acid content were affected significantly by the G × E interaction effect. The best way to improve them would be to plant the appropriate genotypes according to the environment. Figure 7 is a GGE biplot called “Which Won Where,” which is used to select genotypes by the group. The total variation explained by the two principal components (two axes) indicates the degree of fit of the GGE biplot. For the three traits, the degree of fit ranged from 82.74% (the chlorogenic acid content) to 89.02% (the total number of leaves), indicating that the tendency revealed by the GGE biplot was consistent with the actual value.

Each biplot was divided into several parts by rays. Then, the experimental sites were grouped, and the superior genotypes were at the vertex within each environmental group. Six sites were classified in the same group regardless of the trait. The vertex clone for the total number of leaves and the gutta-percha content was clone 4, while the vertex clone for the chlorogenic acid content was clone 16.

## Discussion

To satisfy directional improvement of *E. ulmoides*, many studies have been conducted to understand target traits. For example, genetic linkage maps have been constructed to analyze the quantitative trait loci of growth traits (Li et al., 2014a; Jin et al., 2020). Moreover, *E. ulmoides* genomes have been assembled to guide gutta-percha biosynthesis (Wuyun et al., 2018; Li et al., 2020). Comparative transcriptome analysis has been performed to identify candidate genes related to chlorogenic acid biosynthesis (Ye et al., 2019). Furthermore, researchers have established a probability grading index system to evaluate the economic traits of *E. ulmoides* germplasm (Deng et al., 2021). However, the study of phenotypic trait variations has been rare, a factor that has limited trait improvement (Wu et al., 2013; Pont et al., 2020) of *E. ulmoides*. In this study, phenotypic trait variation was divided into the site, genotype, and G × E interaction effects (Jiang et al., 2021; Matsumoto et al., 2021) by using a mixed linear model. There were significant differences in the genotype and site effects for all the studied traits (Table 3), indicating that superior genotype and preferable cultivation area selection were effective (Pei et al., 2020). The genotype effect contributed the most to the phenotypic variations for the total number of leaves, the gutta-percha content, and the tree height. For the other traits, the site effect played a leading role in the phenotypic variation. Hence, these traits could be improved based on the relative importance of the effects that produce better improvement.

Compared with phenotypic correlation, genetic correlation can exactly reflect the relationship between traits because it excludes environmental influence (Chen and Shen, 2005). In this study, there were similar results in genetic and phenotypic correlations among the studied traits, which indicated that the phenotypic correlations were mainly controlled by genetic correlations for the studied traits (Pei et al., 2020). There were significant positive correlations within growth traits or economic traits, while there were weak negative correlations among growth and economic traits (Figure 5). These findings indicated that simultaneous improvement of economic and growth traits was impossible. Fortunately, the gutta-percha, chlorogenic acid, and rutin contents could be improved together to enhance economic performance. Similar results have been reported for growth and wood traits in other species (Ouyang et al., 2017; Gallo et al., 2018; Jin et al., 2019; Pan et al., 2020; Suraj et al., 2021). These findings underscore the difficulty of simultaneously improving traits in different categories (Hong et al., 2014; Ismael et al., 2021). In addition, the development of *E. ulmoides*-based industries presents a directional trend. Therefore, *E. ulmoides* should be improved with respect to breeding targets to gain greater improvement in certain aspects.

*Eucommia ulmoides* is a dioecious plant (Tippo, 1940), and researchers have reported differences between the sexes (Zhao et al., 1996; Wang et al., 1999; Shi et al., 2017). Besides, a sex difference is a distinguishable feature of the genotype effect. It seems that considering the sex effect contributes to *E. ulmoides* improvement. Statistical analysis revealed a significant difference between the sexes for the gutta-percha content, the chlorogenic acid content, the total number of leaves, and tree height (Table 5). Moreover, the gutta-percha content of male trees was higher than that of female trees (Figure 6). However, these data were not consistent with previous studies. Ye et al. (2014) reported a non-significant difference between the sexes for the chlorogenic acid content. Moreover, Wang et al. (1999) indicated that the gutta-percha content of female trees was significantly higher than that of male trees. These discrepancies are probably due to the relatively small amount of experimental material used in studies. Specifically, the experimental material of this study comprised five female and four male *E. ulmoides* clones, and Wang et al. (1999) and Ye et al. (2014) used four to five female and male *E. ulmoides* individuals, respectively. Therefore, this study represents a preliminary analysis of the sex effect on traits. However, the greater number of tested sites in this study has increased the reliability of the results. The next step is to increase the amount of experimental material to verify the results (Wu et al., 2021).

The environmental effect on a trait mainly lies in phenotypic plasticity (Marcatti et al., 2017). There was a difference in phenotypic plasticity among different traits at the same site. Obviously, the coefficient of variation of economic traits was larger than that of growth traits at the same site (Table 6), indicating the stronger phenotypic plasticity of economic traits (Wu et al., 2021). Compared with growth traits, better improvement effects could be obtained for economic traits. In addition, there were great differences in phenotypic plasticity among different sites for the same trait, a phenomenon that reflects the discriminating ability of sites (Frutos et al., 2014). For example, Liuzhi performed better in discriminating genotype for the gutta-percha content (37.29%), while Yixian was inferior (14.75%) (Table 6). A stronger discriminating ability is necessary for an ideal environment (Jiang et al., 2021). It could amplify the differences among genotypes, a factor that contributes to selecting superior genotypes.

The G × E interaction effect refers to the relative performance differences of a genotype among environments (Ramburan et al., 2012). A better understanding of this effect contributes to the implementation of testing and selection in forest tree breeding programs. There were significant differences in the G × E interaction effect for the total number of leaves, the gutta-percha content, and the chlorogenic acid content (Table 3). These traits could be improved by selecting appropriate genotypes in local environments, which is in accordance with the principle of planting according to the environment (De Moraes Goncalves et al., 2013). The G × E interaction effect was further analyzed using a GGE biplot. Coincidentally, the experimental sites were classified in the same sector, indicating that these sites could be treated as a mega-environment. The superior genotypes were clone 16 (for the chlorogenic acid content) and clone 4 (for the total number of leaves and the gutta-percha content) in the experimental sites. The two clones could be candidate genotypes to improve local economic conditions. Other clones were not located in the sector where the experimental sites were located, indicating that they were not suitable for the experimental sites (Jiang et al., 2021).

## Conclusion

To directionally improve the traits of *E. ulmoides*, nine clones were distributed across six sites to reveal the crucial effects of the subdivision on the phenotype. For many traits, the site effect accounted for a larger proportion of the variance, followed by the genotype and G × E interaction effects. There were significant differences among genotypes or cultivation areas for all the traits studied, indicating they could be improved by selecting preferable genotypes or cultivation areas. However, only the gutta-percha content, the total number of leaves, and the chlorogenic acid content were affected significantly by the G × E interaction effect. These traits could also be improved by following the principle of planting proper trees according to the environment. Traits within growth traits or economic traits could be improved simultaneously, while traits among growth and economic traits could not be improved simultaneously. In addition, leaf yield could be improved simultaneously with most traits. It is likely that multi-trait improvement is feasible in the selection of preferable genotypes. Annual mean temperature and annual sunshine duration proved to be the crucial environmental factors that affected the studied traits. They were correlated positively with economic traits and leaf yield but correlated negatively with growth traits. These findings are beneficial for selecting a wider range of preferable cultivation areas. This study has provided preliminary findings regarding the main effects on the traits of *E. ulmoides* and has offered solutions to improve traits directionally. This information could serve as a reference for the trait improvement of other plants.

## Data Availability Statement

The original contributions presented in the study are included in the article/Supplementary Material, further inquiries can be directed to the corresponding author/s.

## Author Contributions

PD contributed to conceptualization, methodology, software, formal analysis, data curation, writing original draft, visualization, and investigation. YW and HY contributed to investigation and data curation. FH contributed to resources. YL, TW, HZ, and YZ contributed to investigation. ZL contributed to conceptualization, methodology, funding acquisition, supervision, writing, reviewing, and editing. All authors read and approved the final version of this manuscript.

## Funding

This study was supported by the Shaanxi Research and Development (R&D) Program (2019NY-012).

## Conflict of Interest

The authors declare that the research was conducted in the absence of any commercial or financial relationships that could be construed as a potential conflict of interest.

## Publisher's Note

All claims expressed in this article are solely those of the authors and do not necessarily represent those of their affiliated organizations, or those of the publisher, the editors and the reviewers. Any product that may be evaluated in this article, or claim that may be made by its manufacturer, is not guaranteed or endorsed by the publisher.
