# Supplementary figures and images for: Phenotypic Trait Subdivision Provides New Sight Into the Directional Improvement of Eucommia ulmoides Oliver
Source: Front Plant Sci. 2022 Apr 8;13:832821. doi: 10.3389/fpls.2022.832821 (PMC9026163; doi:10.3389/fpls.2022.832821)

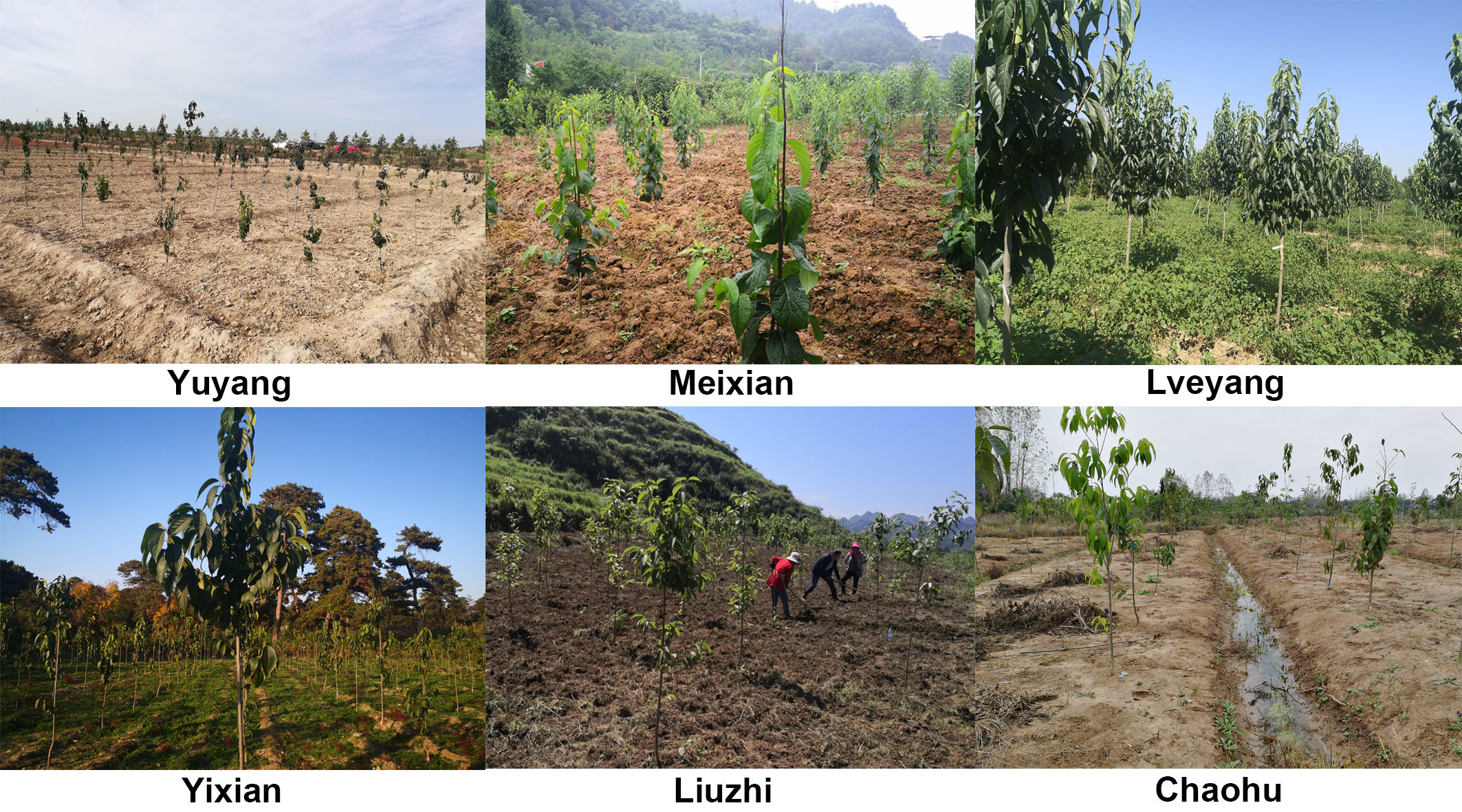

Supplement: Supplementary file 1 [file Image_1.TIF]
