# Supplementary material for: Phenotypic Trait Subdivision Provides New Sight Into the Directional Improvement of Eucommia ulmoides Oliver
Source: Front Plant Sci. 2022 Apr 8;13:832821. doi: 10.3389/fpls.2022.832821 (PMC9026163; doi:10.3389/fpls.2022.832821)
Supplement: Supplementary file 2 [file Table_1.DOCX]

Supplementary Material


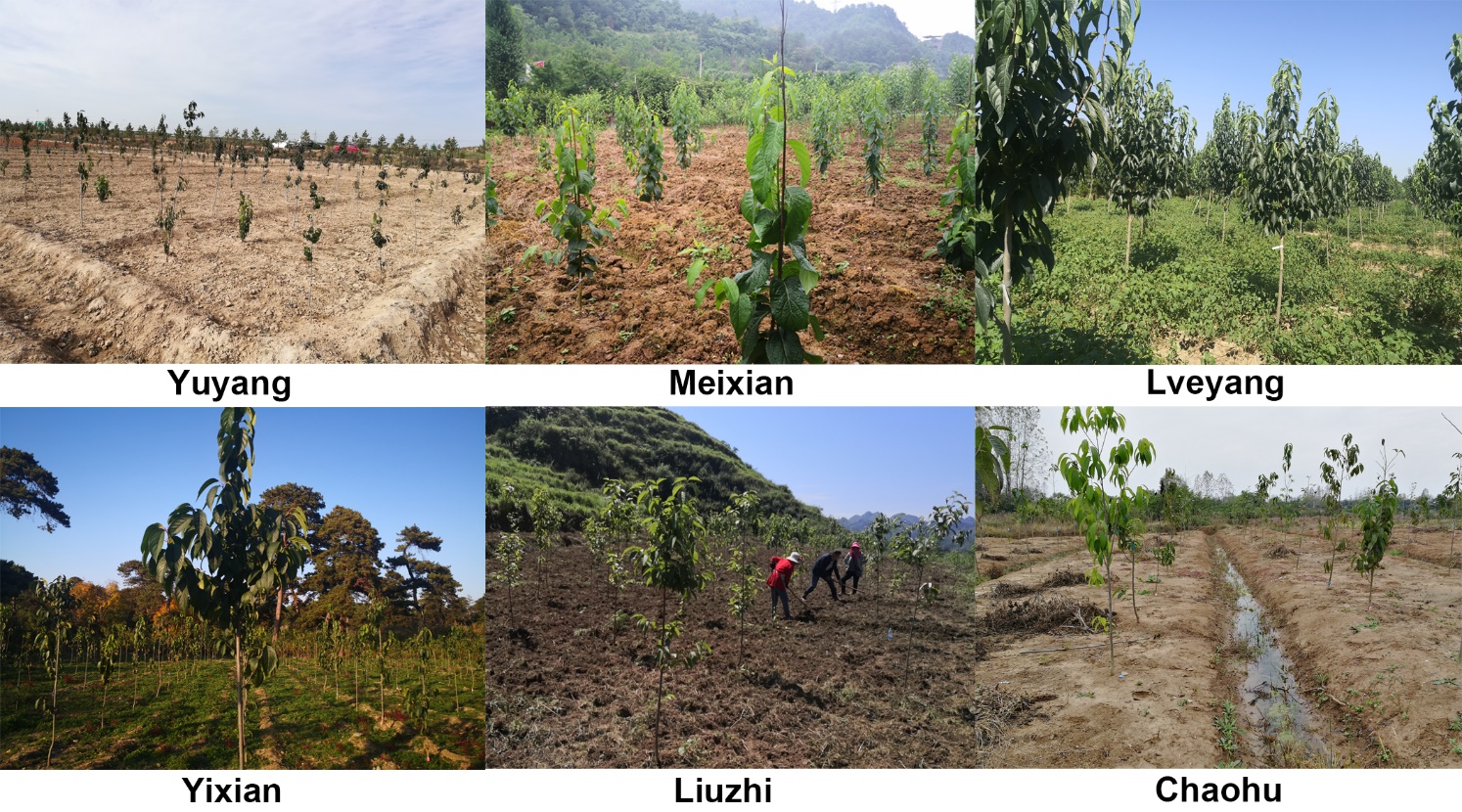


**Supplementary Figure 1.** Growth performance of *Eucommia ulmoides* Oliver in six experimental sites.
